# Epigenetics in Research and Practice

**DOI:** 10.1155/2013/536804

**Published:** 2013-12-22

**Authors:** Debra E. Lyon, Susan G. Dorsey, Leorey Saligan

**Affiliations:** ^1^Virginia Commonwealth University, School of Nursing, Richmond, VA 23298, USA; ^2^University of Maryland School of Nursing, Baltimore, MD 21201, USA; ^3^National Institute of Nursing Research, National Institutes of Health, Bethesda, MD 20892-1506, USA

This special issue focused on the intersection of epigenetics with nursing research and practice. The first paper in this series addresses the role of epigenetic modifications in pain and analgesia response, highlighting the need for future research on epigenomic modification in the development of chronic pain, and summarizes the therapeutic potential to alter epigenetic processes to improve health outcomes. The second studies the epigenetic alterations and an increased frequency of micronuclei in women with fibromyalgia, highlighting a difference in an epigenetic biomarker in participants versus controls. The third paper explored the role of epigenetics in critical illness and the need for clinicians to understand and navigate the novel therapies of the future based on advances in epigenetic science. The fourth paper described the complexity of recruiting participants for epigenetic research and highlighted strategies, including scripts for facilitating the informed consent process. The fifth paper was an insightful review of the epigenetic mechanisms that may contribute to the biological response to trauma and risk for posttraumatic stress disorder. The sixth paper discussed the state of the science in understanding measures of cellular aging in depression.

*Debra  E.  Lyon* *Debra  E.  Lyon* *Susan  G.  Dorsey* *Susan  G.  Dorsey* *Leorey  Saligan* *Leorey  Saligan*

